# A case of endoscopic ultrasonography-guided choledochoduodenostomy for malignant distal bile duct obstruction with upside-down stomach

**DOI:** 10.1055/a-2307-7805

**Published:** 2024-05-17

**Authors:** Takuya Takayanagi, Yusuke Sekino, Kota Ueno, Shota Matsumoto, Noriki Kasuga, Hajime Nagase

**Affiliations:** 1 84178 Department of Gastroenterology, Yokohama Rosai Hospital, Yokohama, Japan

Endoscopic ultrasonography-guided biliary drainage (EUS-BD) is increasingly being used as a drainage technique in cases where endoscopic retrograde cholangiopancreatography (ERCP) cannot be performed. However, in patients with a massive esophageal hiatal hernia, this poses a risk of mediastinitis due to thoracic puncture. Upside-down stomach, first described in 1926 as type IV diaphragmatic hiatal hernia, is a rare condition in which a large portion of the stomach migrates into the thoracic cavity due to organoaxial rotation 1 . There have been reports of ERCP in patients with upside-down stomach 2 3 but none of EUS-BD. This is the first report of EUS-BD for distal bile duct obstruction in a patient with pancreatic head cancer and upside-down stomach.

An 84-year-old woman presented to our hospital with the chief complaint of weight loss. Computed tomography revealed a 35-mm tumor in the pancreatic head, and obstruction of the distal bile duct and duodenum by the tumor was suspected ( Fig. 1 ). The patient also had an esophageal hiatal hernia of the upside-down stomach type, and almost all of her stomach had prolapsed into the thoracic cavity ( Fig. 2 , Fig. 3 ). We decided to attempt endoscopic ultrasonography-guided choledochoduodenostomy (EUS-CDS) along with duodenal stent placement in the papillary region.

**Fig. 1:**
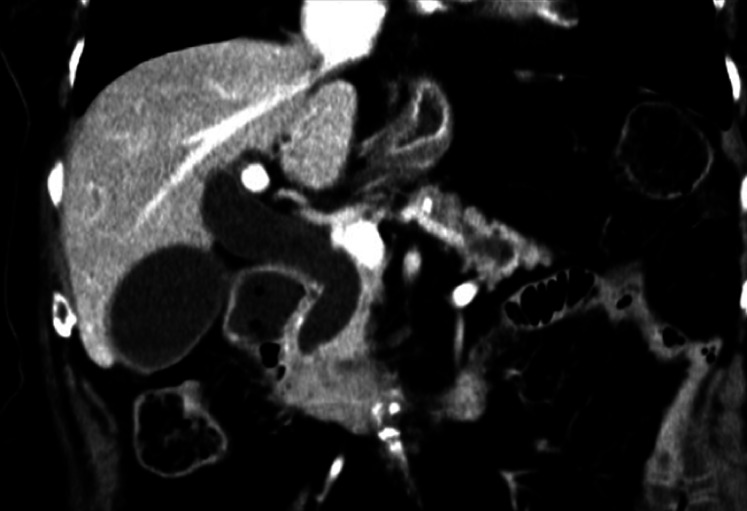
Computed tomography revealed a 35-mm tumor in the pancreatic head, and obstruction of the distal bile duct and duodenum by the tumor was suspected.

**Fig. 2:**
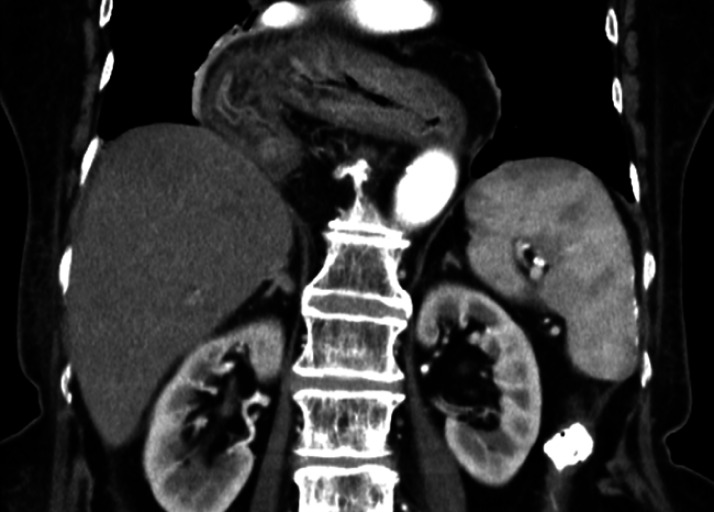
The patient also had a esophageal hiatal hernia of the upside-down stomach type, and almost all of her stomach had prolapsed into the thoracic cavity.

**Fig. 3:**
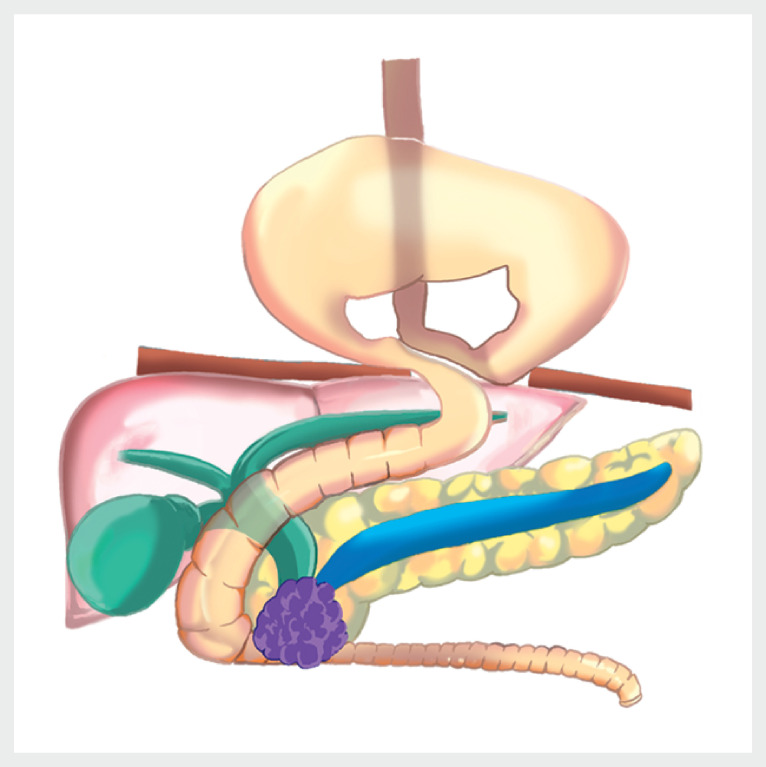
Schema of this case.

A forward-viewing scope was advanced to the duodenum, and a stiff-type guidewire was advanced to the jejunum ( Fig. 4 ). The guidewire was left in place, and the endoscope was removed while the loop for the upside-down stomach was released. A convex endoscope (UCT-260; Olympus, Tokyo, Japan) was then advanced over the wire to the esophagogastric junction. The contrast catheter was advanced, and the endoscope was successfully maneuvered around it to the duodenum. Thereafter, EUS-CDS was performed, and a covered self-expanding metal stent (ZEO STENT, 8 × 60 mm; Zeon Medical Inc., Tokyo, Japan) was placed successfully ( Video 1 , Fig. 5 ). This case demonstrates that EUS-BD can be safely performed for malignant distal bile duct obstruction with upside-down stomach if EUS-CDS is used.

**Fig. 4:**
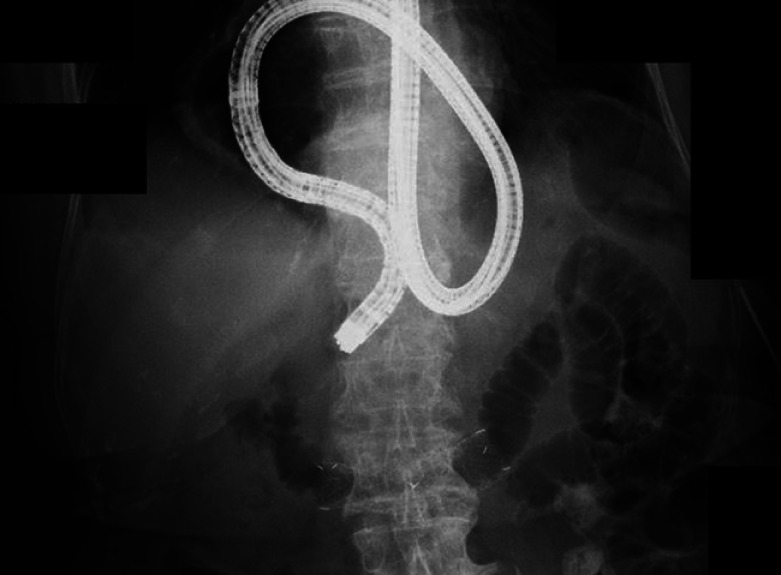
A forward-viewing scope was advanced to the duodenum, and a stiff-type guidewire was advanced to the jejunum. The guidewire was left in place, and the endoscope was removed while the upside-down stomach loop was released.

**Fig. 5:**
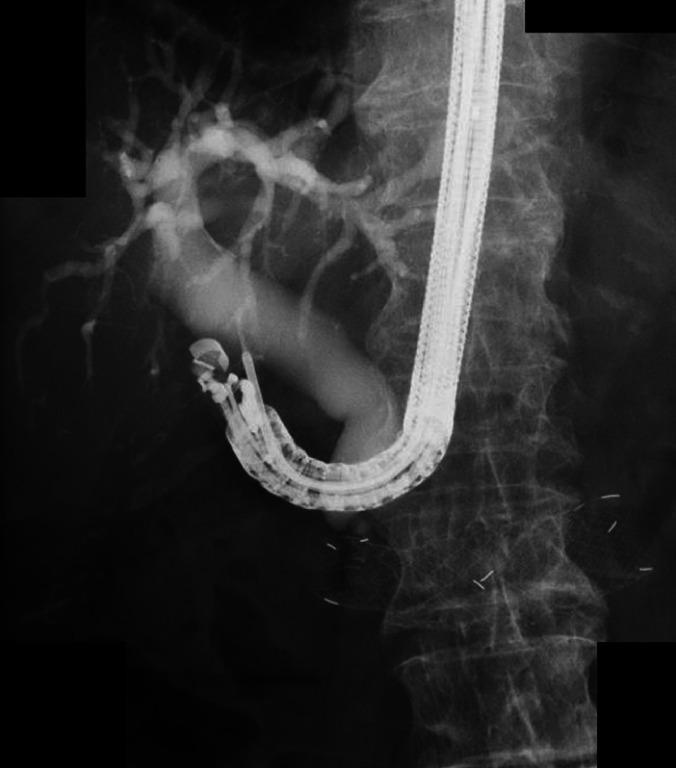
A convex endoscope was successfully maneuvered around it to the duodenum. Thereafter, EUS-CDS was performed, and a covered self-expanding metal stent was placed successfully.

Endoscopic ultrasonography-guided choledochoduodenostomy for malignant distal bile duct obstruction with upside-down stomach.Video 1

Endoscopy_UCTN_Code_TTT_1AS_2AH

## Notes

Endoscopy E-Videos https://eref.thieme.de/e-videos
*E-Videos* is an open access online section of the journal *Endoscopy* , reporting on interesting cases and new techniques in gastroenterological endoscopy. All papers include a high-quality video and are published with a Creative Commons CC-BY license. Endoscopy E-Videos qualify for HINARI discounts and waivers and eligibility is automatically checked during the submission process. We grant 100% waivers to articles whose corresponding authors are based in Group A countries and 50% waivers to those who are based in Group B countries as classified by Research4Life (see: https://www.research4life.org/access/eligibility/ ). This section has its own submission website at https://mc.manuscriptcentral.com/e-videos .
